# MicroRNA-367-3p directly targets RAB23 and inhibits proliferation, migration and invasion of bladder cancer cells and increases cisplatin sensitivity

**DOI:** 10.1007/s00432-023-05484-6

**Published:** 2023-11-08

**Authors:** Xifeng Wei, Yuchen Jiang, Guanghua Yang, Taihao Chang, Guangyu Sun, Shuaiqi Chen, Shangrong Wu, Ranlu Liu

**Affiliations:** 1 Department of Urology, The Second Hospital of Tianjin Medical University & Tianjin Institute of Urology, 23 Pingjiang Road, Hexi District, Tianjin, 300211 China; 2 Department of Urology, People’s Hospital of Ningxia Hui Autonomous Region, Yinchuan, China

**Keywords:** Bladder cancer, MicroRNA-367-3p, RAB23, Proliferation, Cisplatin sensitivity

## Abstract

**Objectives:**

This study investigated the biological role of miR-367-3p upregulation in bladder cancer and verified the mutual relation between miR-367-3p and RAB23.

**Materials and methods:**

Expression levels of miR-367-3p were determined by RT–qPCR in bladder cancer cell lines and human bladder cancer tissues. The effects of miR-367-3p on proliferation, migration and invasion were evaluated by cell colony formation assays, wound healing assays and trans-well assays, respectively. The effects of miR-367-3p and RAB23 on cisplatin sensitivity of bladder cancer cells were assessed by CCK-8 assay. The expression of its target-RAB23 was determined by western blotting in T24, 5637. Plasmids used in dual-luciferase assays were constructed to confirm the action of miR-367-3p on downstream target-RAB23 in T24 cells. And also, the role of miR-367-3p in tumorigenesis was also confirmed in nude mouse models.

**Results:**

The downregulation of miR-367-3p was observed in human bladder cancer tissues. MiR-367-3p downregulation positively correlated with tumor stage and tumor grade. MiR-367-3p overexpression in T24, 5637 cells suppressed the proliferation, migration, and invasion of bladder cancer cells in vitro while decreasing IC50 values under T24 and 5637 cisplatin treatment conditions. RAB23 was shown to be upregulated in bladder cancer tissues and cell lines. MiR-367-3p directly bound to the 3′ UTR of RAB23 in T24 cells. RAB23 was potentially accounted for the aforementioned functions of miR-367-3p. Tumor formation experiments in nude mouse models confirmed that overexpression of miR-367-3p could inhibit tumor growth and invasion in vivo.

**Conclusions:**

miR-367-3p acts as a tumor suppressor in bladder cancer by downregulating RAB23 signaling. We conjecture that miR-367-3p-mediated downregulation of RAB23 expression may be a new therapeutic strategy for bladder cancer treatment.

**Supplementary Information:**

The online version contains supplementary material available at 10.1007/s00432-023-05484-6.

## Introduction

Bladder cancer (BCa) is a major health issue because of its high morbidity and mortality. BCa ranks as the 10th commonest malignancy worldwide (Bray et al. 2018), with an estimated 550,000 annual new cases globally and accounting for about 2.1% of total deaths resulting from cancers (Richters et al. 2020). More than 90% of bladder cancer cases are classified as bladder urothelial carcinomas (BUC) (Veskimae et al. 2019). The prognosis of patients with BUC depends on cancer cells’ invasion and metastatic ability. Although tremendous efforts have been made in surgical techniques and adjuvant chemotherapy, BCa remains a highly prevalent and lethal malignancy (Shirodkar and Lokeshwar 2009). The complex carcinogenic mechanism and molecular targeted therapy of bladder cancer have yet to be fully elucidated. Strategies based on new molecular networks and targets for BCa progression are desperately needed to improve the poor diagnosis and therapies when treating BCa patients.

Chemotherapy is one of the most important treatments for bladder cancer, but some patients still face tumour recurrence, progression and even distant metastases, and chemoresistance is one of the reasons for this, the exact molecular mechanisms of which have not been fully elucidated. Cisplatin has shown good cytotoxicity in a variety of tumours, including bladder cancer and many other solid tumours. Cisplatin exerts its anticancer effects through various mechanisms, but it is now widely believed that cisplatin interacts with purine bases on DNA, forming inter- and intra-strand cross-links that produce DNA damage and ultimately inhibit DNA synthesis and replication (Rabik and Dolan 2007). Although cisplatin has become a mainstay of cancer treatment, its use and effectiveness are limited by two major factors: acquired resistance to cisplatin and severe side effects on normal tissues (Wang and Lippard 2005). However, the molecules associated with the efficacy of cisplatin remain unclear. Therefore, there is a need to further define the underlying mechanisms of cisplatin resistance to enhance the sensitivity of bladder cancer cells to cisplatin and improve cisplatin efficacy.

MicroRNAs (miRNAs) are a class of noncoding RNAs with lengths ranging from 19 to 22 nucleotides, which main function is to bind to 3′-untranslated regions (3′-UTR) of mRNAs complementarily to inhibit translation or mark the mRNAs for degradation (Zhou et al. 2015). Various biological processes, such as cell proliferation, differentiation, and motility, are regulated by miRNA. The dysfunction of miRNAs, which can function as either tumor suppressors or oncogenes, plays vital roles in the tumorigenesis of several cancers, including bladder urothelial carcinoma (Enokida et al. 2016).

MicroRNA-367, whose gene is located on chromosome 4q25, is a member of the miR-302/367 cluster, and targets different genes functions versatilely in diverse neoplastic diseases. MiR-367 has been shown to take a vital part in the tumorigenesis of various types of cancers, such as medulloblastoma (Kaid et al. 2015), pancreatic cancer (Zhu et al. 2015), non-small cell lung cancer (Campayo et al. 2013), and gastric cancer (Bin et al. 2015). MiR-367’s roles and regulatory mechanisms in BUC remain largely unexplored.

RAB23 is one of the Rab GTPase family proteins belonging to the Ras small GTPase superfamily (Smith et al. 2007). Rab GTPases play versatile roles in the proliferation, migration, invasion, and communication of tumor cells (Recchi and Seabra 2012). Recently, numerous studies have indicated that RAB23 is upregulated in multiple types of tumors and takes part in the tumorigenesis of liver cancer, gastric cancer, prostate cancer, and bladder cancer, and is closely concerned with tumor progression (Liu et al. 2007; Hou et al. 2008; Wang and Qin 2018; Ho et al. 2012). These studies indicated RAB23 could act as an oncogene in human cancers. RAB23 expression in normal and cancer tissues was under the influence of genetic and epigenetic factors (Chen et al. 2016). However, the biological functions of RAB23 in human bladder cancers had not been clearly defined.

So far, several studies have focused on splicing factors or miRNAs in RAB23 transcriptional or post-transcriptional regulation. However, whether miR-367-3p can regulate the RAB23 expression and impact the proliferation, invasion, and migration of bladder cancer cells has not yet been elucidated. We conjecture that miR-367-3p-mediated downregulation of RAB23 expression may be a new therapeutic strategy for bladder cancer. To verify this conjecture, bioinformatics and corresponding experiments were employed to probe the molecular mechanisms of miR-367-3p-mediated downregulation in the tumorigenesis and progression of bladder cancers. A dual luciferase reporter assay was used to confirm whether miR-367-3p binds to the 3′ UTR of RAB23 mRNA and affects the tumorigenic activity of bladder cancer cells. We found that miR-367-3p acts as a tumor suppressor in BCa cells, and we also identified RAB23 as its direct target. Meanwhile, we explored the role of miR-367-3p and RAB23 in the treatment of bladder cancer with cisplatin by CCK-8 assay. Our results suggest that miR-367-3p may enhance the sensitivity of cisplatin in bladder cancer cells through RAB23.Our study lays the scientific foundation for enhancing cisplatin treatment of BCa patients through upregulation of miR-367-3p and for the development of new drugs.

## Materials and methods

### Ethics statement

Informed consent was signed by all participants before the experiments. The experiment procedure and protocol were approved by the Ethics Committee of the People’s Hospital of Ningxia Hui Autonomous Region (2021-NZR-034) and complied with the guidelines and principles of the Declaration of Helsinki. The animal experiments were conducted by the Guide for the Care and Use of Laboratory Animals approved by the Animal Care and Use Committee in the Second Hospital of Tianjin Medical University.

### Clinical samples

Forty-two pairs of human bladder cancer tissues and their normal tissues adjacent to cancer were obtained from patients at the People’s Hospital of Ningxia Hui Autonomous Region between Feb 2018 and June 2020. Immediately after resection, all samples were frozen in liquid nitrogen for further examination. The histologic evaluation was confirmed by two seasoned pathologists in our hospital. The histological diagnosis and tumor grades and stages were evaluated based on hematoxylin and eosin-stained cancer sections in line with the World Health Organization (Lopez-Beltran and Montironi 2004) classification guidelines. Tumors were classified into Ta, T1, T2, T3, and T4 based on Union for International Cancer Control (UICC) TNM guidelines (2017).

### Cell culture

The human bladder cancer cell lines (T24, 5637) and immortalized human bladder epithelium (SV-HUC-1) cells were commercially obtained from the Cell Bank of the Chinese Academy of Sciences (Shanghai, China). The cancer cell lines were cultured in 1640/DMEM (BI, Israel) with 10% FBS (GIBCO, USA) and penicillin/streptomycin (100 U/ml and 100 mg/ml, respectively) (Solarbio, China) at 37 °C in a humidified CO_2_ incubator. The SV-HUC-1 cells were cultured in F12K (GIBCO, USA) containing 10% FBS (GIBCO, USA) and penicillin/streptomycin (100 U/ml and 100 mg/ml, respectively) (Solarbio, China).

### Compounds

Cisplatin was purchased from MCE (China). According to the solubility, cisplatin (10 mg) was dissolved in DMF (3.33 ml), to obtain a final concentration of 10 mM. The resulting solution was stored at – 80 °C and away from light for long-term storage.

### Transfection

The RAB23 siRNAs (siRNA-858, siRNA-1186, siRNA-1310), miR-367-3p mimic, miR-367-3p inhibitor and negative control were purchased from GenePharma (Shanghai, China). T24 and 5637 cells were seeded to 40–50% confluence in 6-well plates. The medium was separately replaced with fresh medium containing siRNA, miRNA mimics (30 nM) or inhibitors (30 nM) and RFect as the transfection reagent (named si-RAB23 group, miR-367-3p mimic group and miR-367-3p inhibitor group). Cotransfection was performed on cells using both si-RAB23 and miR-367-3p inhibitor (named si-RAB23 + miR-367-3p inhibitor group). The cells were harvested for subsequent analyses 48 h after transfection. Cells transfection efficiency was explored by qRT–PCR. The sequences are listed in Supplementary Table 1.

### Quantitative real-time PCR

Total RNA was extracted from cells with Total RNA Extraction Kit (Omega, China), and cDNA was synthesized with a Reverse Transcription Kit (Biosharp, China). And qPCR was performed using SYBR Green Master (Roche, USA) in Quantagene q225 PCR System (Kubo Tech, China). The reaction procedure was 95 °C for 3 min at first, and then 40 cycles of 95 °C for 12 s and 62 °C for 40 s. U6 and GAPDH were chosen as reference genes for the normalization. miR-367-3p, U6, RAB23, and GADPH primers were synthesized and purchased from GenePharma (Suzhou, China). Genes relative expression was determined with 2^−ΔΔCt^ method. Specific primer sequences are shown in Supplementary Table 2.

### Immunohistochemistry (IHC)

The protocol for immunohistochemistry was adapted from the previous study with some modifications. Briefly, immunostaining of 4-μm-thick deparaffinized sections derived from tumor specimens with 10% neutral formalin fixation was performed using the SP staining kit (ZSGB-Bio, China). Hydrogen peroxide (0.3%) and normal goat serum pre-blocking were applied to eliminate the endogenous peroxide activity and non-specific binding, respectively. Sections were incubated with RAB23 Polyclonal Antibody (1:1000 dilution; Proteintech, China) for 2 h at room temperature and then incubated with a secondary antibody conjugated with HRP, the staining was developed with DAB + kit (ZSGB-Bio, China) and counterstained with hematoxylin and sections were dehydrated in ethanol before mounting in the end. Negative controls (incubation with normal rabbit immunoglobulin) were performed for each experiment and between steps sections were washed thoroughly with PBS. Double-blinded examination on all staining slides was inspected by seasoned pathologists.

### Dual-luciferase reporter gene assay

TargetScan (https://www.targetscan.org) was taken advantage of for predictions of binding sites between miR-367-3p and RAB23 mRNA. pWT-RAB23 vector and pMut-RAB23 vector were then acquired by cloning the 3′ UTR of RAB23 mRNA and mutated form of possible binding sites into the pmirGLO luciferase vector (GenePharma, China), respectively. mimic NC or miR-367-3p mimic were co-transfected with luciferase reporter vectors pWt-RAB23 or pMut-RAB23, respectively, into bladder cancer cell line T24 in 96-well plates with a pRL-TK vector expressing Renilla luciferase (GenePharma, China) as an internal reference using GP-transfect-Mate (GenePharma, China) by the manufacturer’s instruction. At 24 h post-transfection, the cell lysates were measured for luminescence using the Dual-Luciferase Reporter Assay System (Promega, USA). Normalized data were calculated as the ratio of Renilla/Firefly luciferase readouts.

### Cell counting kit-8 (CCK-8) assay

Cells after transfection were harvested at 48 h, and then suspended in RPMI 1640 (with 10% FBS). The cells (3 × 10^3^ per well) were cultured in 96-well plate for 24 h and then cisplatin with varying final concentration (50, 20, 10, 5, 2, 1, 0.5, 0.2 and 0.1 nM) was added to treat cells. Cells were kept at 37 °C, 5% CO_2_ for 48 h. Using the CCK8 detection kit, 10 μL of CCK8 solution was added to each well. A microplate reader (Eppendorf, Germany) was applied for optical density (OD) value detection at 450 nm. The cisplatin dose that suppressed cell growth by 50% (IC50) was calculated through Prism GraphPad.

### Wound healing assay

Cells (5 × 10^5^ per well) were added into 6-well plate till grew on the plate for 24 h. Scratches were then made with 20 μL pipette tip and RPMI 1640 medium was replaced. Images were taken of the scratches at 0 h, 24 h and 48 h at 100 × final magnification. The area of wound closure was measured. Cell migration rate = (wound closure area at 0 h− wound closure area at 24 h)/wound closure area at 0 h × 100%.

### Transwell invasion assay

The transfected cells were seeded onto the upper chamber of a Transwell coated with 200 mg/mL Matrigel (Corning, USA). These cells were maintained in RPMI 1640 medium and the lower chamber was filled with culture medium supplemented with 10% FBS. The non-migrating cells were scrubbed and removed from the upper surface of the membrane after 24 h. The migrated cells on the lower surface were stained with 1% crystal violet after fixation with 4% paraformaldehyde and counted by ImageJ.

### Colony formation assay

Cells were seeded into six-well plates at a density of 500 cells for T24 and 5637 per well. After being cultured in complete culture medium for 10 days, cells were fixed in 4% methanol for 30 min and stained with 1% crystal violet solution.

### 5-ethynyl-2′-deoxyuridine (EdU) proliferation assay

The bladder cancer cell proliferation was assessed through cell proliferation EdU imaging kit (Abbkine, China). 2 × 10^4^ cells per well with RPMI 1640 (with 10% FBS) and cisplatin (0.5 × IC50) were inoculated into 24-well plates for 48 h. The cells were incubated with EdU for 2 h, fixed with 4% paraformaldehyde for 30 min and permeated with 0.5% tritonX-100 for 20 min. Then, EdU Click-iT reaction mixture was added for 30 min in the dark and then DAPI was added for nuclear staining. Percent of EdU incorporation of T24 and 5637 cells was calculated as total EdU-labeled cells divided by the number of total cells.

### Western blot

The whole extracts from T24 and 5637 cells were prepared using total protein extraction reagent (Boster, China). The examination of the protein expression levels of RAB23 was performed separately using Western blot analysis. The total proteins concentration in the extracts was quantified by the BCA protein assay kit (Solarbio, China). The same amount of each total proteins sample was underwent 12% sodium dodecyl sulphate–polyacrylamide gel electrophoresis (SDS–PAGE). Proteins were treated with 5% skimmed milk after transferred to polyvinylidene fluoride (PVDF) membranes. The membranes were incubated overnight at 4 °C with rabbit polyclonal antibody against RAB23 (1:1000 dilution; Proteintech, China), and GAPDH (1:3000 dilution; Proteintech, China). And then, incubation with anti-rabbit or anti-mouse IgG with horseradish peroxidase conjugate (1:3000 dilution, Proteintech, China) at room temperature for 1 h was performed after three times washing with TBST. Target proteins on PVDF membranes were detected using enhanced chemiluminescence (ECL, Solarbio, China) and detected with the ChemiDoc XRS system (Bio-Rad). The Western blot bands’ densities were quantified using Image J (http://rsbweb.nih.gov/ij/), and GAPDH was used as a reference for normalization.

### Tumor xenograft model

For tumor xenograft experiments, a total of 1 × 10^6^ T24 cells with stable miR-367-3p overexpression or control RNA were injected subcutaneously into the right flank of each 5-week-old male BALB/C nude mouse (*n* = 5 per group). The tumor volumes were measured on days 3, 6, 9, 12, and 15 after transplantation. For in vivo cisplatin sensitivity assay, 1 × 10^6^ T24 cells with stable knockdown RAB23 and blank control were injected subcutaneously. Mice were divided into 4 groups 10 days after transplantation: si-NC + saline group, si-RAB23 + saline group, si-NC + cisplatin 2 mg/kg group and si-RAB23 + cisplatin 2 mg/kg group. Saline and cisplatin were given intraperitoneally at 100 μL starting 10 days after transplantation, every 3 days, and tumour volume was measured 2 h after each injection. On day 25, mice were executed 2 h after injection, and the tumours were removed and weighed. Tumor volume (V, mm^3^) was measured using calipers by determining the length (L, mm) and width (W, mm) of the tumor, where V was calculated as (L × W^2^) × 0.5.

## Results

### miR-367-3p is poorly expressed in bladder cancer and human bladder cancer cell lines

**Fig. 1:**
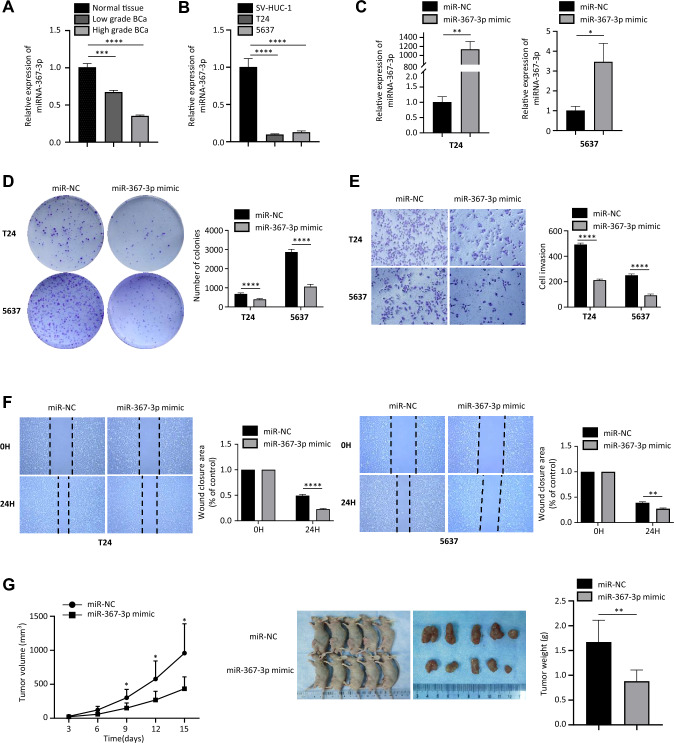
Effect of miR-367-3p overexpression on BCa cell proliferation and invasion. **A** miR-367-3p expression in bladder normal epithelial and BCa tissues. **B** Expression of miR-367-3p in bladder normal epithelial cells SV-HUC-1 and BCa cells T24 and 5637. **C** qRT–PCR analysis of miR-367-3p expression in T24 and 5637 cell lines after miR-367-3p mimic transfection. **D** Effect of overexpression of miR-367-3p on the colony-forming ability of T24 and 5637 cells. **E** Transwell assay to verify the effect of overexpression of miR-367-3p on invasion of T24 and 5637 cells. **F** Scratch assay to assess the effect of miR-367-3p on the migratory ability of T24 and 5637 cells. **G** Effect of overexpression of miR-367-3p on BCa cell tumorigenicity and analysis of the volume of transplanted tumors in each group of nude mice. *P < 0.05, **P < 0.01, ***P < 0.005, ****P < 0.001

To determine the expression level of miR-367-3p in BUC tissues and cell lines, we first inspected its expression level in 42 pairs of BUC tissues and normal tissues adjacent to cancer by qRT–PCR. The clinicopathologic data for the 42 patients are shown in Supplementary Table 3. As shown in Fig. 1A, the data showed the expression of miR-367-3p in BUC tissues decreased significantly compared with that in the paired normal tissues. Meanwhile, miR-367-3p expression was dramatically downregulated in bladder cancer cell lines T24 and 5637 in comparison with normal bladder epithelial cell line SV-HUC-1 (Fig. 1B). We investigated the expression efficiency of miR-367-3p mimics in T24 and 5637 cells by evaluating its expression level by RT–PCR at 48 h post-transfection. The results indicated that miR-367-3p mimics transfection and could boost the expression of miR-367-3p (Fig. 1C). These data have proven that miR-367-3p expression was downregulated in BUC tissues and BCa cell lines, indicating that it may function as a tumor suppressor in bladder cancer.

### miR-367-3p inhibits the proliferation, migration, and invasion of bladder cancer cells

For the purpose of clarifying the biological function of miR 367 3p, cell colony formation assay, wound healing assays and transwell assays were utilized to assess the capabilities of proliferation, migration, and invasion of bladder cancer cells. The cell colony formation assay showed that the proliferation of T24 and 5637 cells was inhibited when transfection with miR-367-3p mimics (Fig. 1D). In addition, the transwell assays showed that the invasion ability of bladder cancer cells in the miR-367-3p mimics group became significantly reduced compared with that in the control group (Fig. 1E). Furthermore, the wound healing assays showed that the migration capability of T24 and 5637 cells was suppressed with miR-367-3p overexpression (Fig. 1F). To clearly elucidate the biological significance of miR-367-3p on tumorigenesis in vivo, T24 cells with stable overexpression of miR-367-3p or miR-NC were injected into the medial thigh of nude mice subcutaneously. The results showed tumor growth got significantly suppressed with miR-367-3p overexpression compared to the controls (Fig. 1G), as evidenced by smaller tumor volume and weight at 2 week post-transplantation (Fig. 1H). These results suggested that miR-367-3p could serve as a tumor suppressor of bladder cancer.

### Overexpression of miR-367-3p sensitizes BCa cells to CDDP

**Fig. 2:**
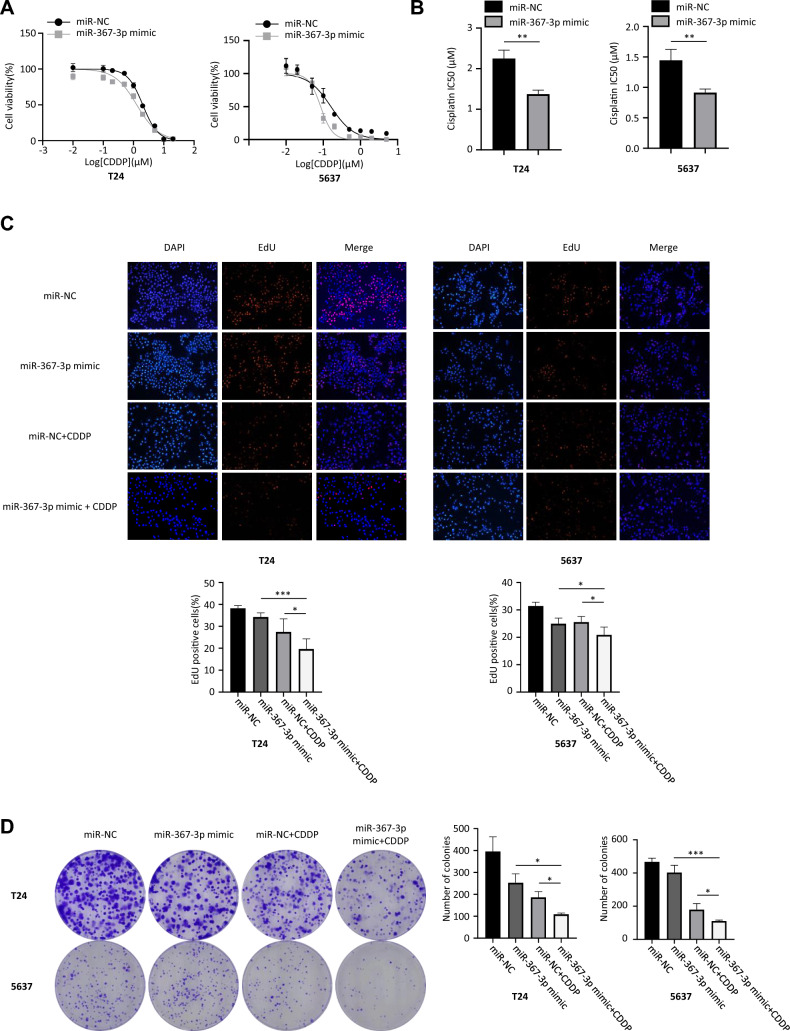
miR-367-3p overexpression enhances cisplatin sensitivity of T24 and 5637 cells in bladder cancer. **A** Dose-inhibition rate curves showing the effect of miR-367-3p overexpression on the chemosensitivity of BCa cells to cisplatin. **B** Effect of upregulated miR-367-3p on IC50 value of T24 and 5637 cells. **C** Effect of upregulation of miR-367-3p and 0.5 × IC50 concentration of CDDP on the proliferative capacity of T24 and 5637 cells as observed by EdU assay. **D** Effect of upregulating miR-367-3p and 0.5 × IC50 concentration of CDDP on the colony-forming ability of T24 and 5637 cells. *P < 0.05, **P < 0.01, ***P < 0.005, ****P < 0.001

We investigated the effect of miR-367-3p on the sensitivity of BCa cells to cisplatin by cell proliferation assays. First, we assayed T24 and 5637 cell viability and IC50 values by CCK-8 assay. Our results showed reduced IC50 values and increased sensitivity of bladder cancer cells in the miR-367-3p mimic-treated group compared to the negative control group (Fig. 2A, B). EdU assay and colony formation assay were used to detect the proliferation ability of cells under cisplatin treatment conditions. Our results showed that miR-367-3p deficiency inhibited the viability of T24 and 5637 cells and reduced the number of cell colonies. The miR-367-3p mimic + cisplatin-treated group significantly inhibited cell proliferation compared to the cisplatin alone and miR-367-3p overexpression groups (Fig. 2C, D).

### RAB23 is a direct target of miR-367-3p in bladder cancer cells

**Fig. 3:**
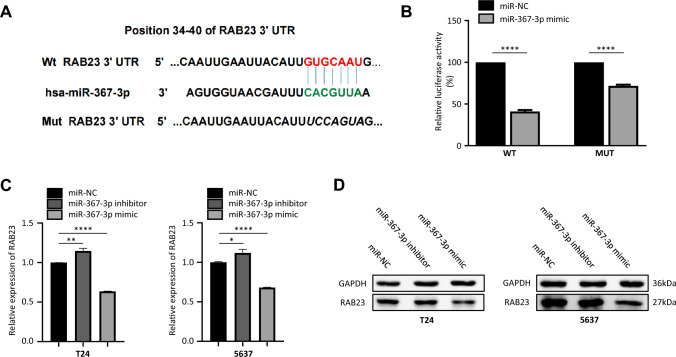
RAB23 is a direct target of miR-367-3p in BCa cells. **A** Bioinformatic prediction of binding sites for miR-367-3p and RAB23. **B** Construction of luciferase reporter system vectors containing wild-type or mutant miR-367-binding sequences. 293 T cells were cotransfected with miR-367-3p or miR-NC with WT or MUT RAB23-3′ UTR. Detection of luciferase activity. **C** qRT–PCR to detect mRNA expression of RAB23 in T24 and 5637 cells transfected with miR-367-3p mimic, miR-367-3p inhibitor and miR-367-3p mimic NC and normalized to GAPDH. **D** Expression of RAB23 protein in T24 and 5637 cells transfected with miR-367-3p was determined by Western blot, and GAPDH was used as an internal control. *P < 0.05, **P < 0.01, ***P < 0.005, ****P < 0.001

It is through the inhibition of target genes that miRNAs mainly function. To find out the underlying direct targets of miR-367-3p in bladder cancer, TargetScan (http://www.targetscan.org/vert_72/) was employed to predict the target genes of miR-367-3p, which might play vital roles in the proliferation, migration, and invasion of bladder cancer (Supplementary Fig. 1). As indicated, binding between 3′ UTR of RAB23 mRNA and miR-367-3p might occur (Fig. 3A). A dual-luciferase reporter gene assay was then conducted to determine whether miR-367-3p directly suppresses the RAB23 expression in bladder cancer cells by the construction of luciferase reporter vectors containing wt or mut sequences of RAB23 3′ UTR. The experimental results revealed that the luciferase activity was markedly depressed after miR-367-3p mimic transfection in comparison with the control. However, the reduction was abolished when the binding site between miR-367-3p and 3′ UTR of RAB23 mRNA got mutated (Fig. 3B). In addition, real-time PCR analysis indicated a significant decrease of RAB23 mRNA in miR-367-3p mimics compared with miR-NC group in T24 and 5637 cells (Fig. 3C). Western blot results revealed RAB23 protein expression significantly decreased with miR-367-3p mimic transfection in comparison with that in the negative control group, suggesting ectopic expression of miR-367-3p can suppress the endogenous RAB23 protein expression (Fig. 3D). Obviously, a negative correlation between miR-367-3p and RAB23 expression levels exists in bladder cancer samples. As suggested, miR-367-3p in bladder cancer cell lines directly targets RAB23 mRNA.

### RAB23 expression is upregulated in bladder cancer

**Fig. 4:**
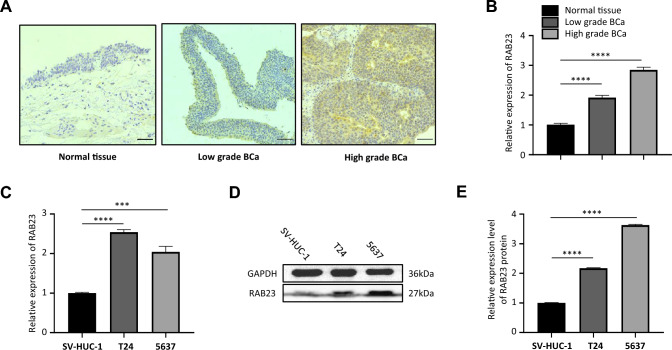
Expression of RAB23 in normal human bladder tissue, bladder cancer tissue and bladder cancer cell lines. **A** Immunohistochemical assessment of RAB23 expression in normal bladder epithelium, low-grade bladder cancer and high-grade bladder cancer. **B** qRT–PCR assay to observe the mRNA expression of RAB23 in human bladder cancer and normal bladder urothelial tissues. **C** qRT–qPCR to detect the relative expression levels of RAB23 in SV-HUC-1 and bladder cancer cell lines (T24,5637). **D** and **E** Western blot assessment of RAB23 expression in normal bladder epithelial cells and bladder cancer cell lines. *P < 0.05, **P < 0.01, ***P < 0.005, ****P < 0.001

RAB23 is a RABGTPase family protein that plays an important role in the progression of vesicular transport and endocytosis of lysosomes. Recent studies have revealed that RAB23 is involved in several biological processes in malignant tumors, and a related study found that RAB23 has a significant function in the progression of cisplatin resistance in ovarian cancer (Zhang et al. 2018). We investigated the expression in normal bladder epithelial tissue and bladder tumor tissue by immunohistochemistry. The immunohistochemical data indicated there was much more RAB23 protein in the bladder cancer samples relative to that in normal bladder urothelial samples (Fig. 4A). We verified that the RAB23 mRNA was higher in the bladder cancer tissues than that in normal tissues adjacent to cancer, as presented in Fig. 4A. Moreover, similar results were obtained in BCa cell lines, as shown in Fig. 4C, D, the RAB23 mRNA and protein expression levels were higher in T24 and 5637 cells than that in normal bladder epithelial cell line SV-HUC-1.

### RAB23 downregulation enhances cisplatin sensitivity of BCa cells

**Fig. 5:**
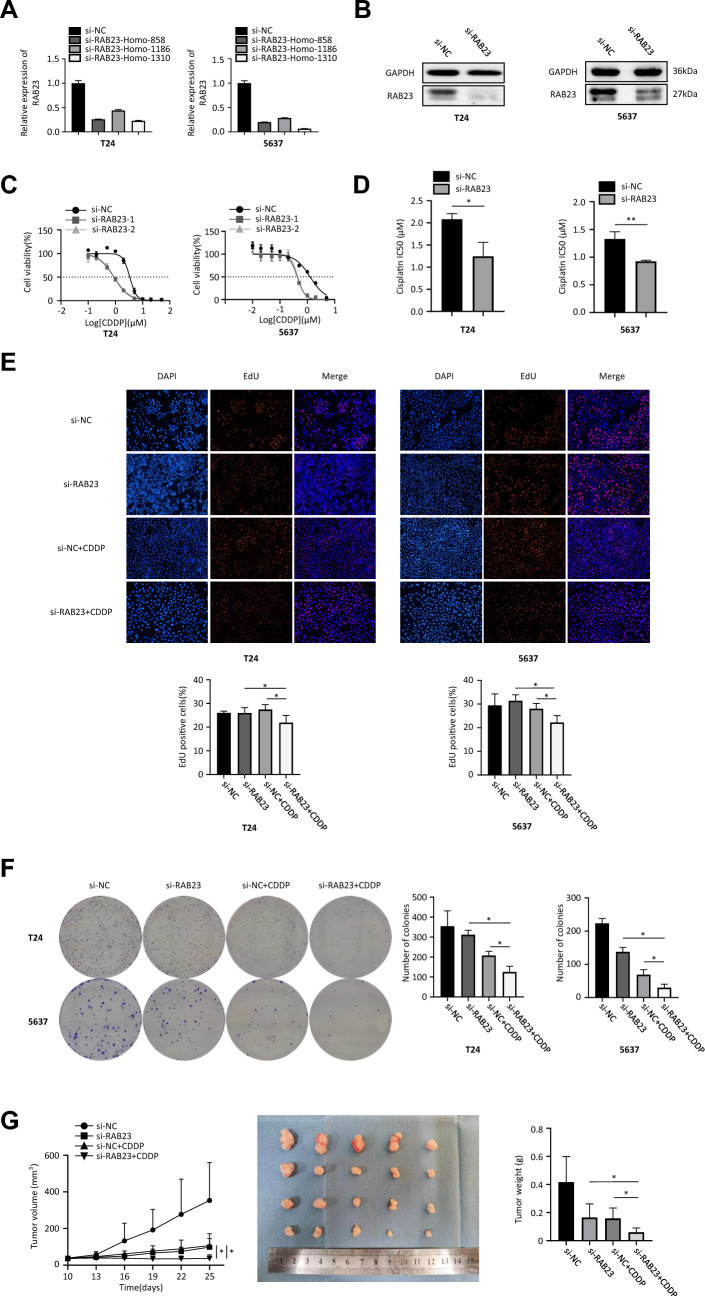
Down-regulation of RAB23 enhances cisplatin sensitivity of BCa cells in bladder cancer. **A** qRT–PCR analysis of RAB23 mRNA expression in T24 and 5637 cell lines transfected with si-RAB23-1, si-RAB23-2 and si-NC. **B** Expression of RAB23 protein in T24 and 5637 cells transfected with si-RAB23 was determined by Western blot, and GAPDH was used as an internal control. **C** Dose inhibition rate curves showing the effect of RAB23 on the chemosensitivity of T24 and 5637 cells to cisplatin. **D** Effect of downregulated RAB23 on IC50 value of T24 and 5637 cells. **E** Effect of down-regulation of RAB23 and 0.5 × IC50 concentration of CDDP on the proliferative capacity of T24 and 5637 cells as observed by EdU assay. **F** Effect of down-regulation of RAB23 and 0.5 × IC50 concentration of CDDP on the colony-forming ability of T24 and 5637 cells. **G** Effect of knockdown of RAB23 and CDDP on the volume and quality of subcutaneous tumor formation in mice. *P < 0.05, **P < 0.01, ***P < 0.005, ****P < 0.001

We transfected cells with small interfering RNAs targeting different loci of RAB23 and found that knockdown of RAB23 by small interfering RNAs targeting the 1310 locus was most pronounced in T24 and 5637 cells (Fig. 5A). In addition, the transfection efficiency was further verified by Western blot (Fig. 5B). We then assessed the effect of RAB23 on malignant proliferation of bladder cancer cells under cisplatin treatment conditions by clone formation, CCK-8 and EdU assays. The results of the CCK-8 assay showed that the inhibitory effect of cisplatin on the viability of T24 and 5637 cells was significantly enhanced after RAB23 downregulation (Fig. 5C) and the cell IC50 values were significantly reduced (Fig. 5D). We then performed an EdU assay to verify the effect of RAB23 on the proliferation of bladder cancer cells after cisplatin treatment. The experimental results showed that there was no statistical difference between the RAB23 knockdown group and the blank group. In contrast, the EdU positivity rate of the RAB23 knockout group after cisplatin treatment was significantly lower than that of the blank group after the addition of 0.5 × IC50 concentration of cisplatin treatment (Fig. 5E). The results of clone formation assay showed that the number of colonies formed by T24 and 5637 cells in the si-RNA + cisplatin treated group was reduced compared to the other treated groups (Fig. 5F). Subcutaneous tumorigenic assays in mice showed that intraperitoneal injection of cisplatin alone or knockdown of RAB23 resulted in significantly lower tumor weight and volume compared to control mice. si-RAB23 + CDDP group showed the most significant tumor suppression compared to other groups. This is consistent with the results of in vitro experiments, indicating that knockdown of RAB23 enhances cisplatin sensitivity in bladder cancer (Fig. 5G).

### miR-367-3p reduces cisplatin sensitivity in tumor cells reversed by RAB23

**Fig. 6:**
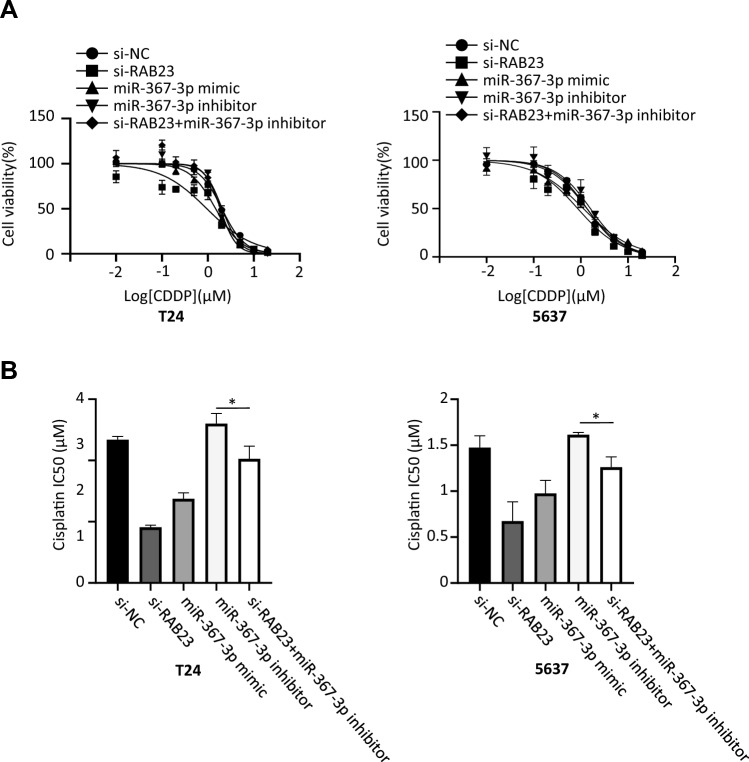
Reduced cisplatin cell sensitivity by miR-367-3p can be reversed by RAB23. **A** Impacts of upregulated miR-367-3p and downregulated RAB23 on growth inhibition rate of T24 and 5637 cells at different concentrations of cisplatin. **B** Impacts of upregulated miR-367-3p and downregulated RAB23 on IC50 value of T24 and 5637 cells. *P < 0.05, **P < 0.01, ***P < 0.005, ****P < 0.001

Analysis of CCK-8 assays showed increased growth inhibition and significantly lower IC50 values of CDDP in BCa cells transfected with miR-367-3p mimic or si-RAB23 relative to BCa cells transfected with mimic-NC or si-NC, while the miR-367-3p inhibitor treatment group had no significant effect on IC50 (Fig. 6A, B). In BCa cells transfected with miR-367-3p inhibitor + si-RAB23, the growth inhibition rate and IC50 values of CDDP were not significantly different compared with the si-NC group, but significantly decreased compared with the miR-367-3p inhibitor group. miR-367-3p decreased cisplatin sensitivity of tumor cells can be somewhat reversed by down-regulation of RAB23, which may be an important target for regulating chemoresistance in BCa cells.

### miR-367-3p down-regulates RAB23 expression and inhibits cellular mismatch repair

**Fig. 7:**
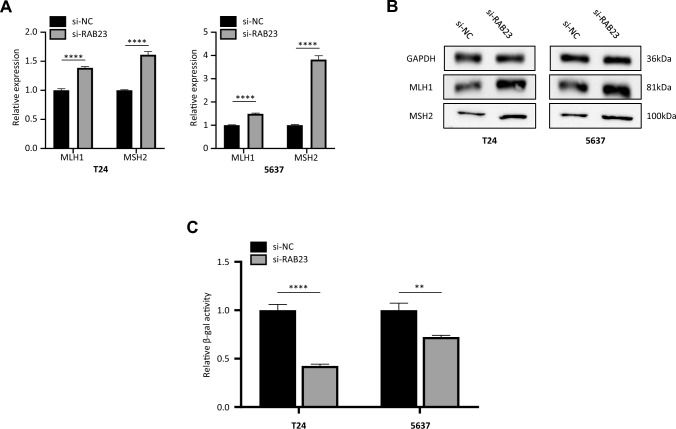
miR-367-3p overexpression downregulates Rab23 expression and inhibits cellular mismatch repair. **A** Detection of MLH1 and MSH2 expression in T24 and 5637 cells transfected with si-RAB23. **B** Expression levels of MLH1 and MSH2 in T24 and 5637 cell lines were examined by Western blot analysis. **C** Effect of RAB23 on cellular MMR. T24,5637 cells stably under-expressing RAB23 were transfected with the pCAR-OF plasmid. The GFP plasmid was also transfected into the cells as an internal control. Relative β-galactosidase activity was normalized by GFP and β-galactosidase activity was determined. *P < 0.05, **P < 0.01, ***P < 0.005, ****P < 0.001

Next, we investigated the effect of miR-367-3p on the mismatch repair-related proteins MLH1 and MSH2. RT–PCR results showed that the mRNA expression levels of MLH1 and MSH2 were significantly increased in T24 and 5637 cells after knockdown of RAB23, with statistical differences compared to negative controls (Fig. 7A). We subsequently examined the changes in MLH1 and MSH2 expression levels by western blot analysis. The experimental results showed that MLH1 and MSH2 were up-regulated in the si-RNA RAB23-treated cells (Fig. 7B). We verified whether knockdown of RAB23 increases cellular MMR activity according to the method mentioned by (Mihaylova et al. 2003; Zhang et al. 2023). The pCAR-OF plasmid, which contains a β-galactosidase gene with a 58-bp out of frame (CA)_29_ insertion sequence at the 5-terminal. Therefore, an increase in β-galactosidase activity can indicate a decrease in MMR functional activity. We measured cellular β-galactosidase activity after transfection of the pCAR-OF plasmid into T24, 5637 cells with stable knockdown of RAB23 and used GFP expression as a normalized control. The results showed that β-galactosidase activity was significantly reduced and cellular DNA MMR activity was increased in bladder cancer cells with knockdown of RAB23 compared with the control group (Fig. 7C). The above experimental results suggest that miR-367-3p down-regulates RAB23 expression and enhances cell mismatch repair, thereby enhancing cisplatin cell sensitivity.

## Discussion

Bladder cancer is the most prevalent malignancy of the urinary system. Although comprehension of the pathogenesis of bladder urothelial carcinoma has improved a lot, the mechanisms of its tumorigenesis are still unclear, discovering and developing novel indicator need to be urgently illustrated for effective bladder cancer diagnosis and treatment. This study sought to elucidate the potential regulatory mechanism of miRNAs, namely, miR-367-3p, on RAB23 gene expression and the effect of miR-367-3p on cisplatin sensitivity in bladder cancer cells.

Amounts of research have revealed that miRNAs participate in tumorigenesis by regulating their target genes. In addition, a series of dysregulated miRNAs have been suggested to take part in the tumorigenesis of bladder cancer (Enokida et al. 2016; Kim et al. 2016). One study identified specific roles of four miRNAs in the progression and metastasis of MIBC and might be viewed as a promising biomarker for MIBC prognosis (Xu et al. 2015). Accumulating evidence has shown that miR-367 overexpression retard tumor progression in several cancers, such as gastric, ovarian, and prostate cancer (Bin et al. 2015; Zheng et al. 2020; Du et al. 2021). These findings have shed light on our study and prompted us to conjecture miR-367-3p might play a vital role in the tumorigenesis of bladder cancers.

MiR-367-3p is a tumor-suppressor gene and takes part in cell proliferation, migration, and apoptosis. As proved by previous studies, miR-367-3p could play a tumor-suppressive role in several cancers. For instance, a previous study has revealed that the expression of miR-367-3p was markedly downregulated in prostate cancer tissues and PC3 cells (Du et al. 2021). However, miR-367-3p expression and its function in BUC tissues get seldom probed. It was for the first time that miR-367-3p expression was demonstrated to be downregulated in BCa cell lines, indicating that miR-367-3p might be a tumor-suppressor gene in BCa. To confirm our speculation, we testified the tumor-suppressive role of miR-367-3p in the proliferation, migration, and invasion of bladder cancer with cell colony formation assay, wound healing assays, and, transwell assays. In line with the findings described above, miR-367-3p expression in BCa patients treated with radical cystectomy was quantified, and the results showed miR-367-3p expression has a negative correlation relationship with disease progression in patients. Our data also suggested that the expression of miR-367-3p did not correlate with other clinicopathological characteristics, such as the age, sex, and tumor size, revealing that miR-367-3p might not take part in the growth of bladder cancer (Supplementary Table 3). However, the expression of miR-367-3p correlated with the differentiation level and TNM stage of bladder cancer, suggesting that miR-367-3p may be involved in the differentiation and progression of bladder cancer.

Cisplatin-based chemotherapy is the primary regimen for the treatment of bladder cancer. Growing evidence suggests that aberrant miRNA expression is highly correlated with the development of drug resistance. In this study, the preliminary effects of miR-367-3p mimic, cisplatin and the combination of miR-367-3p mimic with cisplatin on bladder cancer cell viability were mainly confirmed by CCK-8 assays. Our results show that miR-367-3p mimic successfully increased the sensitivity of cisplatin to bladder cancer cells and decreased cell survival. This is consistent with previous findings in ovarian cancer cells. In this study, Zhang et al. induced apoptosis by upregulating miR-367-3p expression, thereby impairing cisplatin resistance in ovarian cancer cells (Zhang 2021).

We found RAB23, one member of the RAB GTPases family, might be a direct target of miR-367-3p in BUC cells. RAB23 expression is a prominent indicator of the occurrence and progression of many tumors. However, different conclusions were drawn from different studies, and the exact mechanism is still beyond reach. It is reported that RAB23 take a vital part in the tumorigenesis of several cancer types, such as hepatocellular cancer, squamous cell carcinoma, and prostate cancer (Liu et al. 2007; Jian et al. 2016; Chang et al. 2017). RAB23 might regulate the proliferation, invasion, and migration of tumor cells, which have been suggested as a potential therapeutic target for prostate cancer treatment (Chang et al. 2017). In addition, upregulated RAB23 expression is observed in bladder cancer specimens and malignant cell growth and invasion capacity get enhanced via the NF-κB pathway (Jiang et al. 2016). RAB23 has become a promising anti-cancer therapeutic target given the fact that it might function as an oncogene and its high expression in a variety of cancer cells.

In this research, RAB23 expression was found to be upregulated dramatically in BUC tissues and cell lines compared with expression in normal bladder tissues and cell lines. The RAB23 expression level was higher in tumors with a high-rank grade than that with a low-rank grade. This characteristic of RAB23 expression in BUC was similar to that in gastric cancer, where RAB23 expression levels have a positive correlation with tumor grading (Hou et al. 2008). A previous study has demonstrated that RAB23 overexpression can promote proliferation and invasion of bladder cancer cells (Jiang et al. 2016). These findings suggested the oncogenic roles of RAB23 in human cancers.

About its potential mechanism, the bioinformatic analysis predicted that RAB23 was a potential target of miR-367-3p. Dual luciferase reporter assays were conducted to confirm the physical binding between miR-367-3p and 3′ UTR of RAB23 mRNA. The data revealed that miR-367-3p could bind to the 3′ UTR of RAB23 mRNA, indicating that RAB23 acted as a downstream target of miR-367-3p in BCa cells. Results also indicated overexpression of miR-367-3p inhibited the tumorigenesis of bladder cancer by repressing RAB23 expression. These data show that miR-367-3p served as a tumor suppressor in urothelial bladder carcinoma (UBC). These findings also suggested RAB23 might act as a mediator of miR-367-3p function on cellular proliferation, migration, and invasion. Meanwhile, our results showed that down-regulation of RAB23 increased the sensitivity of bladder cancer cells to cisplatin and decreased cell survival. These results suggest that miR-367-3p may reduce cell sensitivity to cisplatin by inhibiting the expression of RAB23.

DNA damage repair is a key component in the development of drug resistance in cells. Among the different DNA damage repair (DDR) pathways, the main ones currently associated with drug resistance are nucleotide excision repair (NER) and mis-match repair (MMR) (Rocha et al. 2018). Our study found that the T24, 5637 cell line, which stably knocked down RAB23, showed a decrease in β-galactosidase activity after transfection with the pCAR-OF plasmid, suggesting an increase in the functional activity of the MMR. The main MMR-related proteins include human mutS homolog 2 (MSH2) and human mutL homolog 1 (MLH1), both of which are associated with cisplatin resistance. Our study showed that increased cellular cisplatin sensitivity after transfection with miR-367-3p mimic may be associated with increased expression of MLH1 and MSH2, which is consistent with previous studies. In a study by Li et al. MLH1 overexpression in tumour cells significantly increased cellular sensitivity to cisplatin and enhanced apoptosis (Li et al. 2018). In addition, in muscle-infiltrating bladder cancer cells, knockdown of MSH2 reduced cellular sensitivity to cisplatin, and low MSH2 expression was associated with lower survival in patients treated with cisplatin (Henrique et al. 2019).

However, more researches are in desperate need of clarifying the downregulation effects of RAB23 by miR-367-3p on cell proliferation, invasion, and anticancer drug resistance in other cancer cell lines aiming to fully illuminate the role of RAB23 in tumorigenesis.

## Conclusions

In summary, our data demonstrated for the first time that miR-367-3p functions as a cancer suppressor in UBC by downregulating the tumor oncogene RAB23. miR-367-3p overexpression enhances the sensitivity of bladder cancer cells to cisplatin, for which RAB23 may be a target. In addition, present study provides strong evidence showing that miR-367-3p could be a novel potential therapeutic target in BUC treatment. In our integrated analysis, RAB23 was a richly expressed gene in BCa cells. Functionally, RAB23 knockdown suppressed the proliferation, invasion, and migration of BUC cells. All in all, the present findings underlined the prominent function of the miR-367-3p/RAB23 signaling regulatory axis in the tumorigenesis of BUC, thereby laid the foundation for BUC-targeted therapy, cisplatin and its combination.

### Supplementary Information

Below is the link to the electronic supplementary material.

Supplementary file1 (DOCX 464 KB)

## Data Availability

All data supporting the findings of this study are included in this published article and supplementary materials.
