# Supplementary material for: MicroRNA-367-3p directly targets RAB23 and inhibits proliferation, migration and invasion of bladder cancer cells and increases cisplatin sensitivity
Source: J Cancer Res Clin Oncol. 2023 Nov 8;149(20):17807–21. doi: 10.1007/s00432-023-05484-6 (PMC10725407; doi:10.1007/s00432-023-05484-6)

Supplementary Table 1. siRNA for transfection

| Gene | Sequences(5' to 3') |
| --- | --- |
| Negative control | sense 5'-UUC UCC GAA CGU GUC ACG UTT-3'  antisense 5'-ACG UGA CAC GUU CGG AGA ATT-3' |
| RAB23-Homo-858 | GGAGGAUUUGAUGCAAUUTT  AAUUGCAUCAAAUUCCUCCTT |
| RAB23-Homo-1186 | GCUGAGGAUCCAGAACUAATT  UUAGUUCUGGAUCCUCAGCTT |
| RAB23-Homo-1310 | CCAACAAACAAAGGACCAATT  UUGGUCCUUUGUUUGUUGGTT |
| GAPDH Positive control | sense 5'-UGACCUCAACUACAUGGUUTT-3'  antisense 5'-AACCAUGUAGUUGAGGUCATT-3' |
| hsa-mir-367-3p inhibitor | UCACCAUUGCUAAAGUGCAAUU |
| hsa-mir-367-3p mimics | AAUUGCACUUUAGCAAUGGUGA  ACCAUUGCUAAAGUGCAAUUUU |
| MircoRNA inhibitor N.C. | CAGUACUUUUGUGUAGUACAA |

Supplementary Table 2. Primer sequences used for qRT-PCR

| Primers | Forward(5'-3') | Reverse(5'-3') |
| --- | --- | --- |
| RAB23 | GGTGGAAGTCACTCCGGTC | GGTCCTTTGTTTGTTGGGTCTA |
| miR-367-3p | CCAGATTGGAATTGCACTTTAGC | TATGGTTGTTCACGACTCCTTCAC |
| U6 | CGCTTCGGCAGCACATATAC | TTCACGAATTTGCGTGTCATC |
| GAPDH | CAGGAGGCATTGCTGATGAT | GAAGGCTGGGGCTCATTT |

Supplementary Table 3. Correlation between the expression of miR-367-3p and clinicopathological characteristics of the BUC patients

| Parameters | n | Relative miR-367-3p expression | |
| --- | --- | --- | --- |
|  |  | median | p-Value |
| Paraneoplastic tissues | 42 | 0.594 | 0.019 |
| Carcinoma tissue | 42 | 0.493 |  |
| Age(years) |  |  |  |
| <60 | 15 | 0.586 | 0.429 |
| ≥60 | 27 | 0.452 |  |
| Sex |  |  |  |
| Male | 31 | 0.580 | 0.262 |
| Female | 11 | 0.368 |  |
| Tumor size(cm) |  |  |  |
| <3 | 27 | 0.531 | 0.802 |
| ≥3 | 15 | 0.432 |  |
| Tumor stage |  |  |  |
| T1 | 25 | 0.668 | 0.000 |
| T2-T4 | 17 | 0.327 |  |
| Histology grade |  |  |  |
| Low Grade | 19 | 0.67 | 0.001 |
| High Grade | 23 | 0.368 |  |

Supplementary Figure 1. Finding downstream target genes of miR-367-3p by TargetScan.


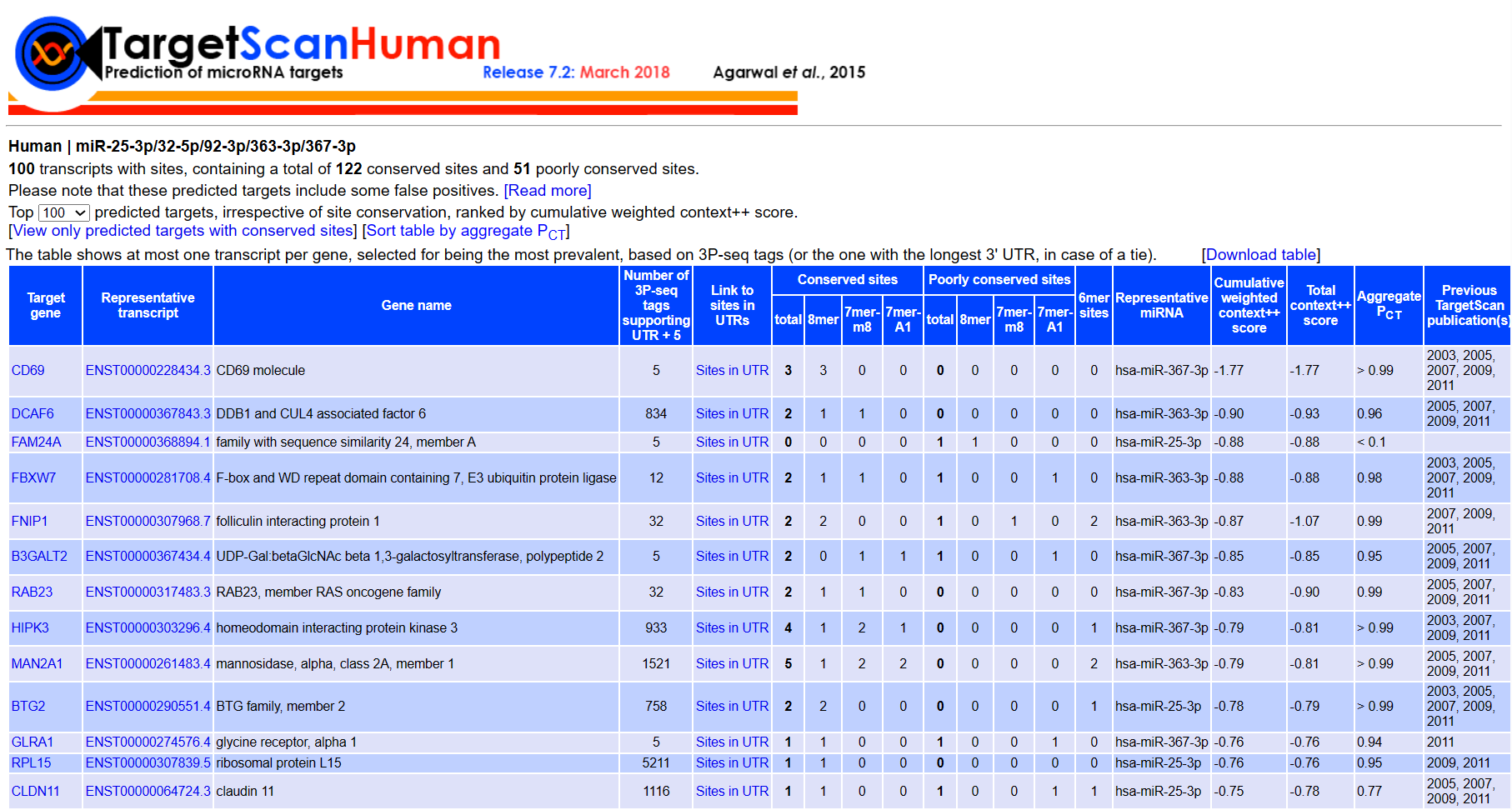

Supplement: Supplementary file 1 — Supplementary file1 (DOCX 464 KB) [file 432_2023_5484_MOESM1_ESM.docx]
